# Guide to the Assessment of Mature Liver Gene Expression in Stem Cell-Derived Hepatocytes

**DOI:** 10.1089/scd.2019.0064

**Published:** 2019-07-16

**Authors:** Mihaela Zabulica, Raghuraman C. Srinivasan, Massoud Vosough, Christina Hammarstedt, Tingting Wu, Roberto Gramignoli, Ewa Ellis, Kristina Kannisto, Alexandra Collin de l'Hortet, Kazuki Takeishi, Alejandro Soto-Gutierrez, Stephen C. Strom

**Affiliations:** ^1^ Division of Pathology, Department of Laboratory Medicine, Karolinska Institutet, Stockholm, Sweden.; ^2^ Royan Institute for Stem Cell Biology, Tehran, Iran.; ^3^ Clinical Science, Intervention and Technology (CLINTEC), Karolinska Institutet, Stockholm, Sweden.; ^4^ Department of Laboratory Medicine, Clinical Research Centre, Karolinska Institutet, Stockholm, Sweden.; ^5^ Department of Pathology, University of Pittsburgh, Pittsburgh, Pennsylvania.

**Keywords:** induced pluripotent stem cells, hepatic differentiation, stem cell-derived hepatocytes, mature liver gene expression, fetal liver gene expression

## Abstract

Differentiation of stem cells to hepatocyte-like cells (HLCs) holds great promise for basic research, drug and toxicological investigations, and clinical applications. There are currently no protocols for the production of HLCs from stem cells, such as embryonic stem cells or induced pluripotent stem cells, that produce fully mature hepatocytes with a wide range of mature hepatic functions. This report describes a standard method to assess the maturation of stem cell-derived HLCs with a moderately high-throughput format, by analysing liver gene expression by quantitative RT-qPCR. This method also provides a robust data set of the expression of 62 genes expressed in normal liver, generated from 17 fetal and 25 mature human livers, so that investigators can quickly and easily compare the expression of these genes in their stem cell-derived HLCs with the values obtained in authentic fetal and mature human liver. The simple methods described in this study will provide a quick and accurate assessment of the efficacy of a differentiation protocol and will help guide the optimization of differentiation conditions.

## Introduction

Liver plays a fundamental role in the body as it performs a wide range of functions, including metabolism, nutrient storage, and detoxification. The definitive treatment for life-threatening liver diseases is orthotopic liver transplantation (OLT). Unfortunately, the demand for transplantable livers is progressively outpacing the supply of donated cadaveric organs, resulting in longer waiting times and increased mortality of prospective transplant recipients [1]. Hepatocyte transplantation has been used as a method to support patients with liver insufficiency and is an alternative to OLT. Hepatocyte transplantation is recognized as a treatment for acquired and inherited hepatic diseases, functioning mainly as a bridge for patients awaiting an available organ [2]. However, their use is limited by the low availability of high-quality donor organs, as well as their low proliferative capacity and inability to sustain mature functions in vitro [3,4]. All these reasons highlight the necessity for the identification of an additional sources of hepatocytes.

A potential alternative to primary hepatocytes could be hepatocyte-like cells (HLC) derived from pluripotent or other stem cells. The generation of induced pluripotent stem cells (iPSC) [5,6], by forced expression of transcription factors, offers a potentially unlimited source of multipotent cells with the capacity to differentiate into other cell types without raising ethical concerns. Early protocols for hepatic differentiation were based on embryoid body formation and spontaneous differentiation with addition of specific growth factors [7–9]. This approach is simple but inefficient, and frequently generates populations with multiple cell types. Later, protocols performed targeted differentiation experiments with growth factors and extracellular substrates identified as important is natural development. The majority of the protocols start with endoderm induction since hepatocytes have an endoderm lineage progenitor. For endoderm induction, several compounds have been shown as essential, such as ActA, Wnt, and BMP4 [10]. In addition to traditional 2D culture, more recent hepatic differentiation protocols include suspension, 3D organoids, or spheroids culture to enhance differentiation [11–13]. These approaches hold great promise for both research and therapeutic applications, drug discovery, and regenerative medicine. HLC generated from pluripotent sources have been used for various applications, such as disease modeling, drug metabolism, virus infection and replication, study of noncoding RNA, and drug-induced liver injury [14]. Most differentiation protocols do not generate fully mature hepatocytes with respect to a diversity of mature hepatic functions, including drug metabolizing capacity, urea cycle activity, or bile acid and lipoprotein synthesis and excretion. Cells generated by most current protocols express genes at levels characteristic of fetal liver and display a more fetal-like phenotype [15].

Many published reports do not compare gene expression in their stem cell-derived hepatocytes to authentic human hepatocytes or human liver, and in the publications that do, the reports are frequently restricted to one or only a few human cases of authentic human hepatocytes or human liver tissue. With many different differentiation protocols, different methods to assess hepatic function and different liver cases as positive controls, it is impossible to compare data from one publication to another and equally difficult to determine how the level of gene expression in the HLC compared to the values that would be observed in normal human liver. The field would be served by a standard method to assess liver gene expression coupled with a robust data set generated from a number of human livers that is available to all researchers so that they can quickly and easily compare the level of gene expression in their sample to that in normal human liver.

In this study, we present quantitative RT-qPCR data on the mRNA expression of more than 60 genes, normally expressed in liver, normalized to an internal control, in a sample set of 17 fetal and 25 mature livers. In addition, we demonstrate how the expression profile in iPSC-derived HLCs from our own laboratory and HLCs commercially available from three companies compared to the standard liver data set. The data were produced with commercially available gene expression assays, making it possible for any laboratory to use these methods and the included data set as a guide to the assessment of HLC maturation.

## Methods

### Collection of liver tissues

All procedures followed were in accordance with the ethical standards of the responsible committees on human experimentation (institutional and national) and with the Declaration of Helsinki 1975, as revised in 2000–2005. All postnatal human liver tissues were collected following Ethical and Institutional Guidelines at the University of Pittsburgh, IRB approval 0411142. Organ donors were tested, and were negative, for hepatitis viruses B and C, and human immunodeficiency virus (HIV). Fetal liver tissues were obtained under protocol, IRB PR010020037, University of Pittsburgh, from tissue donations after selective, induced abortions. The fetal age was estimated by standard clinical parameters and the tissue was kept in Eagle's minimum Essential medium (EMEM) (Lonza, Walkersville, MD), and transported on ice. The postnatal group is composed of liver specimens derived from patients undergoing scheduled liver resection performed for neoplasia, or different reasons. Residual tissue not needed for diagnostic purposes after hepatic resection was transported to the laboratory from the operating rooms on ice in EMEM (Lonza) within 90 min of removal.

### IPSC-derived HLCs

IPSC-derived HLCs used in this study were generated in our laboratory. In addition, cells were purchased from three commercial sources, Reprocell, Cellartis, and Cellular Dynamics International, and labeled A, B, and C, respectively, in the graphs. Differentiation of the commercial cells was conducted in the supplier's laboratory under their specific conditions. When the differentiation protocols were complete, the supplier lysed the cells in TRIzol and shipped the samples on dry ice.

#### Organoid (ORG) hepatocyte-like cells

##### Cell culture

Hepatic fibroblasts were isolated from a 9-month old OTC deficient individual. Cells were cultured in DMEM GlutaMax (Life Technologies), supplemented with 10% heat-inactivated fetal bovine serum (Life Technologies), 100 U/mL penicillin/streptomycin (Life Technologies), and 1% nonessential amino acids (NEAA, Cat. Number 11140; Gibson by Life Technologies). Culture incubation conditions were 37°C and 5% CO_2_. The passaging interval was 4–5 days using TrypLE (Life Technologies).

Before differentiation, iPSC were maintained on Vitronectin (Life Technologies)-coated plates in Essential 8 Basal Medium (Life Technologies). Cells were passaged mechanically every 4–6 days.

##### Somatic cell reprogramming

Fibroblasts were reprogrammed into iPSC using CytoTune-iPS 2.0 Sendai Reprogramming Kit (Invitrogen, Life Technologies) according to manufacturer's instructions. IPSC clones were characterized based on gene expression and protein levels.

##### Differentiation of iPSC into definitive endoderm

In brief, 10^5^ iPSC were seeded in each well of a Matrigel-coated six-well plate and cultured for 20 h in Essential 8 Basal Medium containing 10 μM Rock inhibitor (StemCell Technologies). The protocol for endoderm induction lasted 6 days. Cells were cultured in RPMI GlutaMax medium (Life Technologies), supplemented with 1% B27 (Life Technologies), 1% NEAA (Cat. Number 11140; Gibco by Life Technologies), 100 U/mL penicillin/streptomycin (Life Technologies), and 50 ng/mL ActA (PeproTech). In addition, definitive endoderm medium was supplemented with ITS-X (Life Technologies) and KnockOut Serum Replacement (KOSR) (Life Technologies) at a concentration of 0.1% the first day, 1% the second day, and 2.5% the next 2 days of endoderm induction. Medium was also supplemented with 2.5 μM CHIR (Stem Gent) on the first day. Differentiation continued 2 more days with GlutaMax medium (Life Technologies), 1% NEAA (Cat. Number 11140; Gibco by Life Technologies), 100 U/mL penicillin/streptomycin (Life Technologies), 1% B27 (Life Technologies), 10 ng/μL basic FGF (PeproTech), and 20 ng/μL BMP4 (PeproTech).

##### Organoid formation

Cells were harvested on day 7 with Accutase (Sigma) and 5 × 10^4^ cells were suspended in 50 μL of Matrigel (BD) and plated in ultralow attachment 24-well plates (CORNING) as described for the generation of organoids, without the addition of forskolin and with 5% RSPO1 conditioned medium instead of 10% [16], for the first 4 days postendoderm induction.

##### Hepatic differentiation media

From day 11 until day 28, hepatic differentiation was accomplished, in part, as described in provisional patent application (PCT/US2018/018032) in medium which consisted of 45% Ham's F12 Nutrient Mix medium, 45% DMEM GlutaMax medium, 10% KOSR, and as modified as described here and in cited references, 1% DMSO, 1% NEAA (Cat. Number 11140; Gibco by Life Technologies), 100 U penicillin/streptomycin, 20 ng/mL HGF, 5 mM rifampicin, 10 μM hydrocortisone 21-hemisuccinate [17], 0.1 mM ascorbic acid, 10 μM lithocholic acid [18], 7.18 μM linoleic acid, 10 μM vitamin K2 [18], 10 ng/mL human growth hormone, 0.1 mM dexamethasone, 10 ng/mL oncostatin M [17] for the last 3 days, and differentiation medium was supplemented with 1 mM 8-Bromoadenosine 3′,5′-cyclic monophosphate sodium salt [19]. Medium was changed every other day. Three independent experiments were performed for organoid differentiation. Cells obtained from the commercial sources were described as the best available at the time of purchase, and only one HLC sample was obtained from each source.

### Total RNA extraction and quantitative PCR

Total RNA was extracted by disruption of tissue or cells in TRIzol reagent (Life Technologies) followed by separation of RNA from the water phase either by precipitation with isopropanol or using the Pure Link RNA Mini Kit (Life Technologies). RNA samples were separated on agarose gels containing GelRed Nucleic Acid Stain (Biotium), and the integrity of 18/28S ribosomal RNA was visually assessed. Synthesis of cDNA was carried out with 1 μg of individual total RNA using the High Capacity Reverse Transcriptase Kit (Life Technologies) according to manufacturer's instructions. The cDNA was then diluted 1:10 with nucleotide/RNAse free water. The reaction mix was consisted of 2 μL cDNA input, 5 μL TaqMan Universal PCR Master Mix (Applied Biosystems), 0.5 μL TaqMan assay, and 2.5 μL water. Quantitative expression analysis was performed with TaqMan gene expression assays (Supplementary Table S2 for a complete list of TaqMan assays) using a StepOnePlus system (Applied Biosystems, Life Technologies). The thermal cycle started with incubation for 2 min at 50°C, followed by 10 min at 95°C and finally 40 cycles of 15 s at 95°C and 1 min at 60°C. The cycle threshold was set to 0.082963 in all experiments. This threshold was well within the linear range of all amplification curves. Reactions were run in duplicate or triplicate, with human Cyclophilin A (*PPIA*) mRNA as an endogenous control in all experiments. Calculation of relative levels of expression were done according to the comparative Ct-method [20] as follows: 2^(−ΔCt)^, where ΔCt = *Ct* gene of interest − *Ct* internal control Cyclophilin A. This generates the values that are presented in the graphs. *Ct* values for the gene of interest 35 or higher were considered as unreliable, while those “undetectable” by the PCR machine were set as *Ct* equal to 40. In the gene expression graphs, a gray area has been annotated where the gene expression is so low as to be unreliable. This area has been calculated based on *Ct* equal or higher than 35 and endogenous *C*t 21.47, which was the average for all samples used in the study.

## Results

**FIG. 1.**
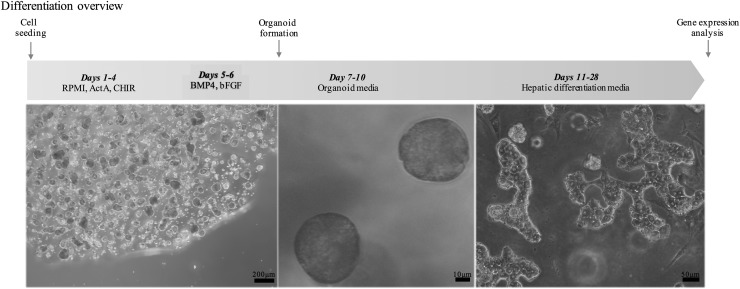
Schematic representation of the hepatic differentiation protocol used for ORG HLC and representative pictures of the cells produced. All images were taken at the end of differentiation experiment. Image on the *right panel* was taken by allowing the HLC to explant from the 3D spheroids and attach, in 2D culture, and was only for photographic and morphological analysis. HLC, hepatocyte-like cell; ORG, organoid.

The demographics of the fetal and mature donors analyzed in this study are presented in Supplementary Table S1. Fetal gestational ages are provided in weeks or days. Of the postnatal liver samples, only 1 case was obtained from a donor less than 10 years of age, and that case, tissue from a 5.5-year-old male showed a gene expression profile that was well within the other mature liver samples analyzed. For this reason, we describe the tissues samples of this postnatal group as representing mature human liver. The information on the donor tissues and the TaqMan assays used for the gene expression analysis are presented in Supplementary Tables S1 and S2, respectively. An overview of the differentiation protocol as described in the methods is presented in Fig. 1 with representative pictures of the different stages. The photo of final hepatic differentiation was taken by allowing the HLC to explant from the 3D spheroids and attach, in 2D culture, and was for photographic and morphological analysis only. The data on gene expression in the tables were generated from the organoid cells directly.

### Liver-specific plasma proteins, metabolic, and phase II gene expression

**FIG. 2.**
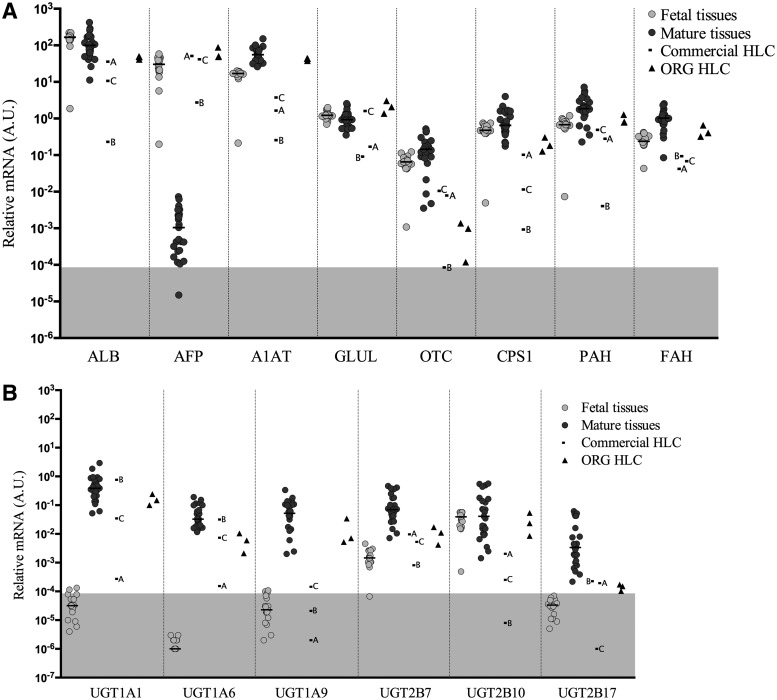
Gene expression levels of liver-specific plasma proteins and metabolic enzymes **(A)** and Phase II conjugation genes **(B)** normalized to endogenous control Cyclophilin A in fetal tissues, mature tissues, commercial HLC, and ORG HLC. *Horizontal* bars represent median values. *Gray* highlighted areas represent unreliable levels of expression.

Data are presented in Fig. 2 through six display relative levels of gene expression, that is, the expression of the gene of interest compared to the internal control Cyclophilin A as indicated in the methods. Calculation of relative levels of expression was done according to the comparative Ct-method [20] as follows: 2^(−ΔCt)^, where ΔCt = *Ct* gene of interest − *Ct* internal control Cyclophilin A. The calculations for all 62 genes for all the tissues and cells analyzed are presented in Supplementary Table S3. In each of the figures, a gray area at the bottom of the graph has been annotated to show where the gene expression is so low as to be unreliable. This area has been calculated from a Ct for the internal control, *PPIA* = 21.47, which is the average Ct for *PPIA* from all samples, and Ct for the gene of interest of 35, 2^(−CT)^, where ΔCt = *Ct* 35 − *Ct* 21.47. This corresponds to a value of 0.000085, and from and below this number, the gray area is highlighted.

All gene names, the abbreviations used in the article and figures for the gene names, and the TaqMan assay reagents used in the analysis are provided in Supplementary Table S2. The entire Excel spreadsheet containing the gene expression data from all the liver cases are provided in Supplementary Table S3.

In Fig. 2A, the expression of albumin (*ALB*) and alpha-fetoprotein (*AFP*), often used as specific indicators of hepatic maturity, is presented. No difference in *ALB* expression was observed between the fetal and mature liver samples. As expected, *AFP* expression was much lower in the mature liver group compared to the fetal. Regarding HLCs, organoid (ORG) HLC as wells as HLC from companies A and C were in the range of the expression of mature liver tissues, while company B was lower. All HLC expressed *AFP* in the range expressed in the fetal samples. Fetal and mature liver samples expressed similar levels of the genes encoding alpha 1-antitrypsin (*A1AT*; *SERPINA1*), glutamate-ammonia ligase (also known as glutamine synthase, *GLUL*), carbamoyl-phosphate synthase 1 (*CPS1*), phenylalanine hydroxylase (*PAH*), and fumarylacetoacetate hydrolase (*FAH*). ORG HLC expressed these genes at mature levels, while the commercial HLCs were frequently lower. Ornithine carbamoyltransferase (*OTC*) expression was generally expressed at higher levels in postnatal tissues, only HLCs A and C reached the lowest levels found in the mature liver samples.

Uridine diphosphate glucoronosyltransferases, enzymes located in the membrane of the endoplasmic reticulum of hepatocytes, are involved in phase II metabolism and catalyze the conjugation of lipophilic molecules with glucuronide to facilitate excretion from the body. Six phase II genes were analyzed (Fig. 2B) and they displayed similar patterns, with lower expression in the fetal tissues than the mature liver counterparts, with the exception of *UGT2B10* where fetal and mature liver gene expression was overlapping. Nearly all the fetal samples for *UGT1A1*, *UGT1A6*, *UGT1A9*, and *UGT2B17* fall in or near the gray-highlighted area, meaning that the expression of the above genes is undetectable or unreliable. Most HLC express the UGT genes at levels higher than those in fetal liver and frequently within the range observed in mature liver.

### Expression of Cytochrome P450 enzymes

**FIG. 3.**
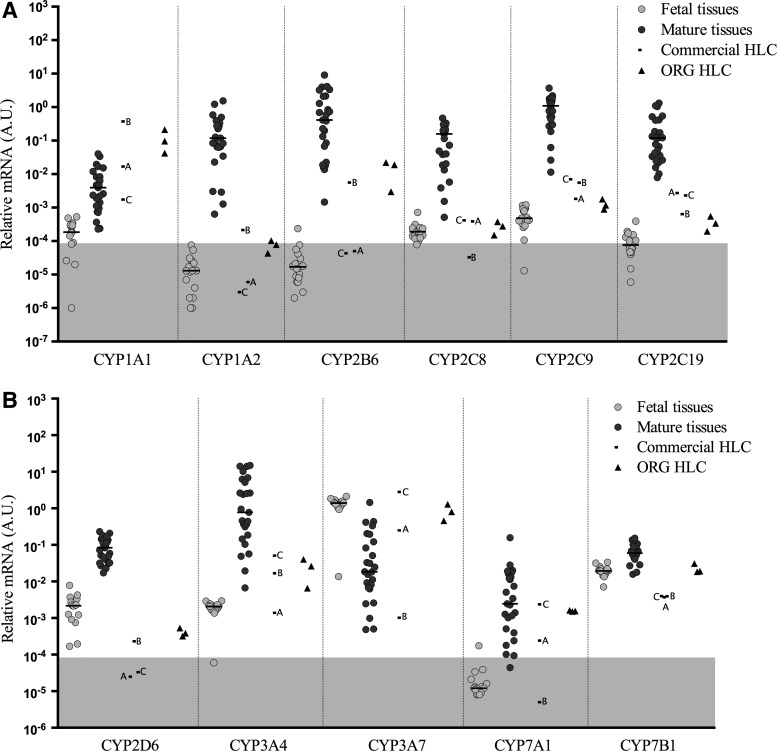
Gene expression levels of cytochrome P450 genes **(A, B)** normalized to endogenous control Cyclophilin A in fetal tissues, mature tissues, commercial HLC, and ORG HLC. *Horizontal* bars represent median values. *Gray* highlighted areas represent unreliable levels of expression.

Human Cytochrome P450 (CYP) genes are involved in the metabolism of thousands of endogenous and exogenous compounds. Thus, they perform central role in the function of hepatocytes. Among the CYPs included in this study, the expression in fetal tissues was lower compared to mature liver cases, except for *CYP3A7*, which is known to be the predominant form of *CYP3A* in the fetal liver (Fig. 3A, B). The relative mRNA levels in the mature liver group ranged over a scale of 10^4^ revealing the very large variation in expression known to exist for CYPs in human liver tissue.

As shown in Fig. 3A, *CYP1A1* levels in ORG HLC were above those usually found in mature liver samples, while commercial samples A and C were in the range observed in the mature liver samples. With the exception of *CYP1A1*, which is expressed in a wide range of tissues, and whose expression profile is not restricted to liver, ORG HLC and those commercially obtained, displayed expression of CYP genes more similar to fetal liver or values in-between fetal and mature liver values. Specifically, all HLCs produced levels of *CYP1A2* and *CYP2C8* in the range observed in fetal livers, while the expression of *CYP2C9*, *CYP2C19*, and *CYP2B6* was generally between that observed in fetal and mature liver. Regarding *CYP2D6*, (Fig. 3B) all HLCs failed to reach mature levels. Two other important CYP genes, *CYP3A4* and *CYP3A7*, which are often referred as the adult and fetal *CYP3A* genes, respectively, were analyzed (Fig. 3B). Some commercial HLC and ORG HLC display expression of *CYP3A4* in the lower levels of mature hepatic tissue, while still expressing *CYP3A7* at near fetal liver levels. *CYP7A1* and *7B1* are involved in cholesterol metabolism and bile acid synthesis and ORG HLC, and commercial samples A and C expressed levels of *CYP7A1* similar to mature tissues, while sample B was undetectable, as were most of the fetal tissues. The expression of *CYP7B1* in ORG HLC was in the high fetal to low mature liver range, while the expression in all the commercial cells were lower than fetal levels.

### Hepatic and other nuclear factors

**FIG. 4.**
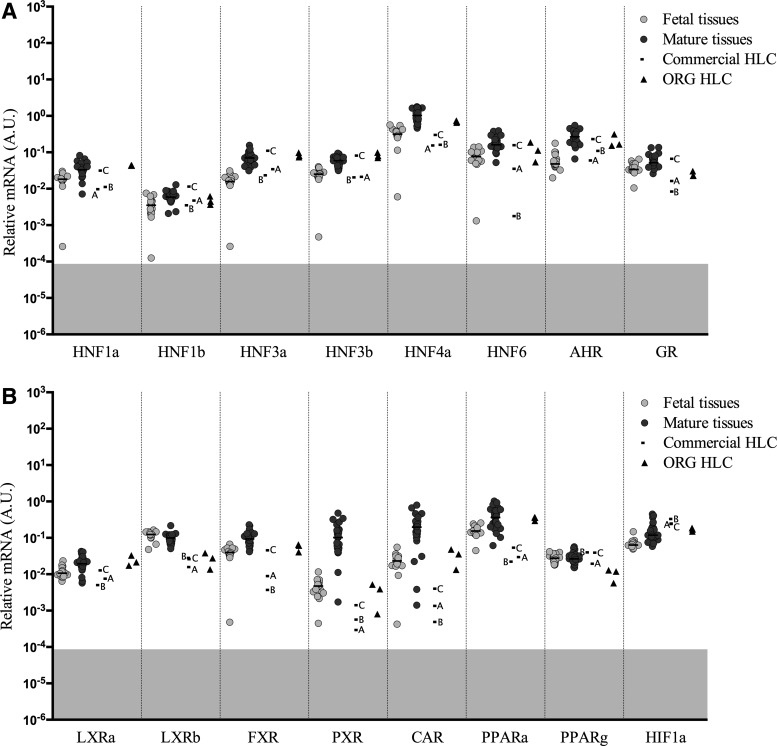
Gene expression levels of hepatic nuclear factors and other transcription factors **(A, B)** normalized to endogenous control Cyclophilin A in fetal tissues, mature tissues, commercial HLC, and ORG HLC. *Horizontal* bars represent median values. *Gray* highlighted areas represent unreliable levels of expression.

Hepatocyte nuclear factors (HNFs) are a group of nuclear hormone receptors expressed predominantly in the liver, but are also present in a number of other tissues. Generally, the median expression of HNF genes in fetal liver was similar, but slightly below the levels measured in the mature liver tissues (Fig. 4A). The ORG HLC expressed *HNF1a*, *HNF1b, HNF3a*, *HNF3b*, *HNF4a*, the aromatic hydrocarbon receptor (*AHR*), and the glucocorticoid receptor (*GR*) transcripts at levels that are in the range of mature liver tissues. Samples from the commercial firms were more variable and generally lower than that observed in the ORG HLC.

Several other transcription factors were analyzed (Fig. 4B), including liver X receptor alpha (*LXRa*) and beta (*LXRb*), hypoxia-inducible 1-alpha (*HIF1a*), farnesoid X receptor (*FXR*), peroxisome proliferator-activated receptor alpha (*PPARa*), and gamma (*PPARg*), where ORG HLC were found to express these genes at levels at the lower end of that observed in mature liver, and there were differences between the expression observed in the ORG HLC and the commercial samples. Pregnane X receptor (*PXR*), constitutive androstane receptor (*CAR*), and transcription factors critical for the expression of the *CYP2B*, *CYP2C*, and *CYP3A* family genes showed a wide range of expression in HLC samples with ORG HLC generally displaying higher levels of expression than the commercial samples. Notably, *PXR* was expressed in ORG HLC at levels normally observed in fetal tissues, while *CAR* expression was generally higher than the median fetal liver levels and was overlapping with the expression of *CAR* observed in some mature liver samples.

### Transporters and multidrug resistance proteins

**FIG. 5.**
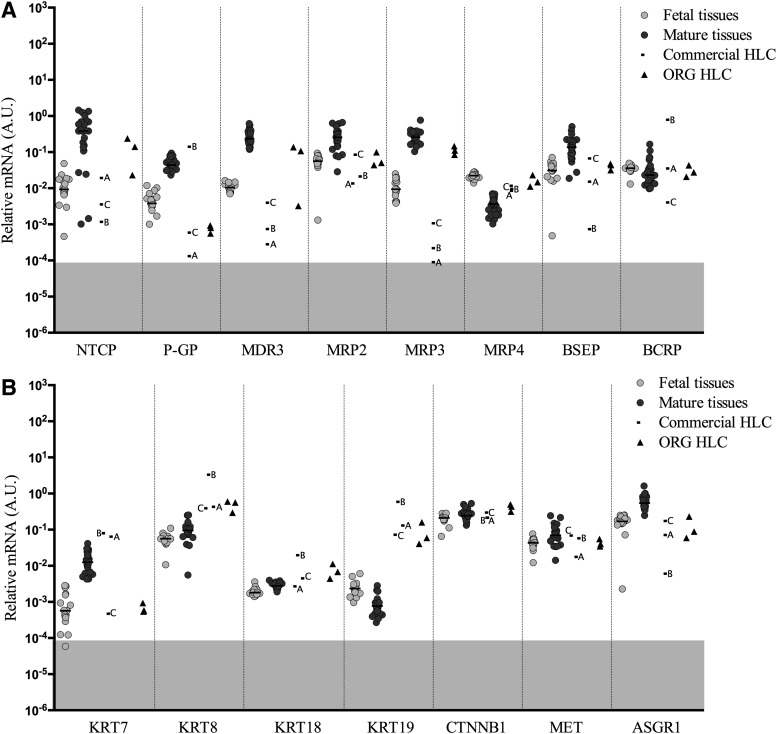
Gene expression levels of transporters and multidrug resistance proteins **(A)** and Cytokeratins, beta catenin, *MET*, and *ASGR1* **(B)** normalized to endogenous control Cyclophilin A in fetal tissues, mature tissues, commercial HLC, and ORG HLC. *Horizontal* bars represent median values. *Gray* highlighted areas represent unreliable levels of expression.

Transporters are proteins involved in the passage of ions, small molecules, or macromolecules through biological membranes. In this study, 8 different transporters and multidrug resistance proteins were analyzed (Fig. 5A). The expression in fetal tissues is generally lower than the mature liver tissues, except from the transcript of multiple drug resistance-associated protein 4 (*MRP4*) which is higher in fetal liver and the transcript of breast cancer resistance protein (*BCRP*) which is similar in fetal and mature liver tissues.

In ORG HLC, *MRP2*, *MRP3*, and *MDR3* are expressed at levels observed in mature liver, while the commercial samples are quite variable. The expression of *P-GP* (also known as *MDR1*) is quite low in the HLC with the exception of commercial sample B. As shown, *BCRP* is not informative since both fetal and mature liver express nearly the same level of this gene. Genes critical for the uptake and excretion of bile acids in/from hepatocytes, Na^+^-taurocholate cotransporting polypeptide (*NTCP*), and bile salt export pump (*BSEP*), respectively, are expressed in ORG HLC at levels higher than most commercial samples and within the range normally observed in mature liver.

### Cytoskeletal and other genes

Cytokeratins (KRT) consist a subfamily of intermediate filaments and are mainly expressed in skin and epithelial cells such as hepatocytes. The most relevant to the liver are *KRT7*, *KRT8*, *KRT18*, and *KRT19* and the magnitude of expression depends on the stage of the organogenesis as well as on the cell subtypes of the liver. In general, HLC displayed higher expression of *KRT8* and *KRT19* than fetal or mature liver, while it was variable for *KRT7* and *KRT18* (Fig. 5B). All HLC displayed expression of catenin beta 1 (*CTNNB1*) and Met proto-oncogene *MET*, the receptor for hepatocyte growth factor, at levels observed in either fetal or mature liver, while the expression of asialoglycoprotein receptor 1 (*ASGR1*), a receptor on hepatocytes that removes targeted proteins from the circulation, generally fell in the fetal range in HLC.

### Pluripotency and early differentiation genes

**FIG. 6.**
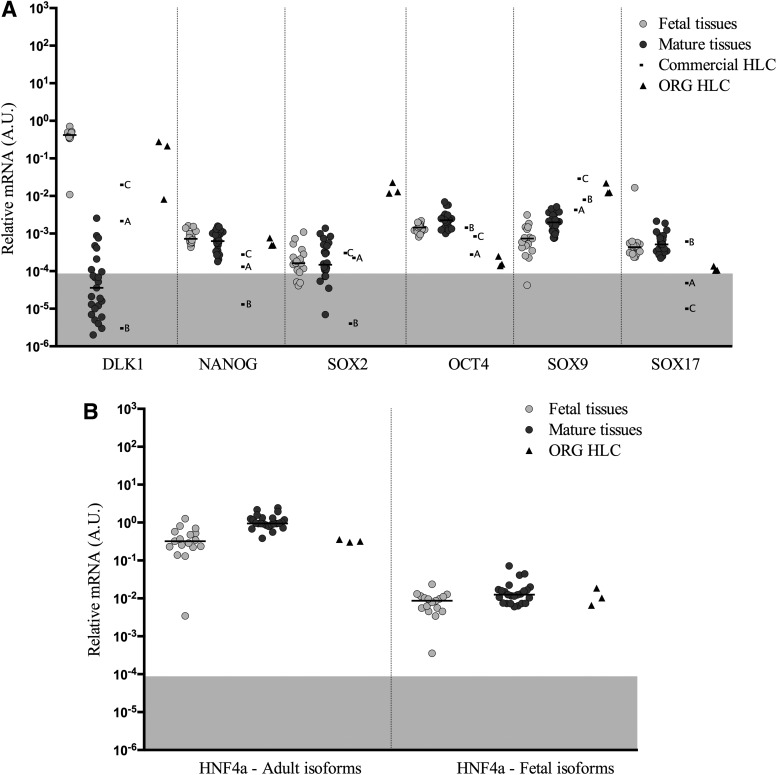
Gene expression levels of pluripontency and stem cell genes normalized to endogenous control, Cyclophilin A in fetal tissues, mature tissues, commercial HLC, and ORG HLC **(A)**. Gene expression levels of *HNF4a* using specific primers to amplify adult isoforms driven by P1 promoter and fetal isoforms driven by P2 promoter, normalized to endogenous control Cyclophilin A in fetal tissues, mature tissues, and ORG HLC **(B)**. *Horizontal* bars represent median values. *Gray* highlighted areas represent unreliable levels of expression. There was not sufficient RNA from the commercial samples to perform this analysis. *Gray* highlighted areas represent unreliable levels of expression.

Differentiation of iPSC into different cell types requires the activation of the tissue-specific genes and the repression of the pluripotency genes; therefore, genes associated with pluripotency and early development were analyzed (Fig. 6). The most common markers of pluripotency are (*NANOG*), sex determining region Y-box 2 (*SOX2*), and octamer-binding transcription factor 4 (*OCT4*), which are expressed at low, but similar levels in the fetal and mature liver tissues were analyzed. In HLC, the level of these transcripts varied considerably between the different samples with no consistent pattern. Delta like non-canonical Notch ligand 1 (*DLK1*) expression was very high in fetal liver, compared to mature liver tissues and HLC generally expressed this gene at levels between fetal and mature liver. Expression of SRY-box 9 (*SOX9*) is generally higher in HLC samples compared to either fetal or mature liver, while an endodermal gene, SRY-box17 (*SOX17*), is generally lower in HLC samples.

### *HNF4a* differential transcripts

*HNF4a* plays an important role in the liver development. The gene encodes different isoforms originating from different promoters and alternative splicing. Specific primers were used to amplify transcripts driven by the adult (P1) or fetal (P2) isoforms. Fetal tissues showed a similar level of expression of fetal transcripts and lower levels of adult transcripts than the mature samples (Fig. 6B). ORG HLC displayed P2-driven transcripts in the range of fetal and mature tissues, but they failed to reach mature levels of P1-driven transcripts (adult form). There was not sufficient material to analyze the commercial samples.

## Discussion

Liver performs a diverse range of functions, including synthesis of numerous molecules, metabolism of nutrients and xenobiotics, elimination of various molecules, secretion, storage and release of metabolic products, as well as neutralization of antigens and microbes from the gut. Given the variety of functions the liver performs, it is easy to understand how mutations or alterations in the expression of critical liver genes leads to liver-based diseases. Currently, the definitive treatment for patients with end-stage liver diseases is OLT. Hepatocyte transplantation, when successful, holds several advantages over OLT, in that it is a minimally invasive procedure and the native liver is not removed, which means that in case of cell graft loss, the patient returns to the previous state. In addition, enzymatic activities or functions diminished or absent in the native liver could be replaced by the transplantation of only a small percentage of the liver mass [21].

However, there are not sufficient numbers of healthy hepatocytes available to accommodate transplants for all the individuals who may benefit from this therapy. In addition, the quality of liver tissues available for cellular therapy are frequently not optimal, and the inability of primary hepatocytes to proliferate in vitro and their susceptibility to cryopreservation damage pose additional burdens for the use of primary cells. There is an unmet need for new sources of hepatocytes for the treatment of liver disease.

Recent advances in stem cell research sparked enthusiasm for the generation of HLC from embryonic stem cells or iPSC, with the latter ones perhaps being preferable because of a lack of ethical concerns, as well as the potential for the generation of cells for autologous transplantation that could eliminate the need for immunosuppressive treatment. However, there are several hurdles to implementation, including the risk of tumor formation due to incomplete differentiation, excessive cell culture costs, and the difficulty to scale production to meaningful levels.

An equally important concern is the efficacy of differentiation protocols to generate mature HLC that would resemble mature hepatocytes. To further optimize differentiation procedures, practical and accurate tools are required to assess the maturity of stem cell-derived HLC. In this study, we report the expression of more than 60 hepatic, pluripotency, and developmental genes in 17 fetal and 25 mature livers. A complete description of the experimental procedure is provided here and a complete Excel spreadsheet containing the gene expression data is provided in Supplementary Table S3. If the specific methods presented in this study were followed, including the use of the specific TaqMan primers and probes and the RT-qPCR conditions, it would enable researchers in other laboratories to quickly and accurately assess the level of gene expression of their HLC and compare it to the levels found in these fetal or mature liver tissues. The point of the study was not to provide an optimal differentiation procedure, but rather to provide a mechanism to determine the level of gene expression in a sample from any differentiation protocol from any laboratory, and to provide a rich data set for comparison of the results with the HLC to the gene expression in authentic fetal and mature human liver. In the analysis, cells produced by three commercial firms along with three replicate samples produced in our own laboratory are included to demonstrate how to conduct the analysis and display and interpret the results.

There is a temporal and developmental progression in the expression and activity of the drug metabolizing enzymes in the liver. An analysis of the expression of the genes encoding these enzymes provides information concerning the state of differentiation of HLC and will provide information to determine if the HLC follow a pattern of expression observed during normal liver development. The preponderance of information on the expression of the CYP450 genes is derived from studies of the metabolism of CYP-specific drugs, and while few reports provide RNA data to corroborate the metabolic activity observed, since the CYP450 genes are mainly regulated at the transcriptional level, one can make general correlations between RNA levels and the reported metabolic activity.

There is considerable variability between individuals and even the results in different studies; however, it is generally agreed that there are certain enzymes that are expressed during the fetal period that decrease in expression in the postnatal period. There is a second group with negligible to low expression during the fetal period, which show a burst of activity at birth and increase with age to adult levels, and a third group that have negligible expression in the fetal and even the early neonatal period and develop full mature levels sometimes years after birth. In many of the CYP families, there are age-related differences in the expression of different family members.

Two members of the *CYP1A* family expressed in the liver were examined, *CYP1A1* and *CYP1A2*. *CYP1A1* is expressed in the fetal liver, and metabolic activity is largely absent in adult liver in the absence of inducers of gene expression such as smoking or exposure to other polycyclic aromatic hydrocarbons (mediated by the AHR) [22–25]. The expression of *CYP1A2* is an example of the third group of genes described above where there is little to no expression of drug metabolizing activity attributable to *CYP1A2* during fetal and the early postnatal period, and there is a slow and progressive increase in *CYP1A2* activity after birth to ∼50% of adult levels by 1 year of age [22–24]. A similar pattern of expression and metabolic activity is thought to exist for *CYP2B6* [23,24]. The ORG HLC produced here express RNA for *CYP1A1* and extremely low levels of *CYP1A2*, which would be characteristic of a fetal phenotype. However, *CYP2B6* expression far exceeds that observed in fetal liver and approaches the lower end of mature gene expression.

There are four members of the *CYP3A* family, *CYP3A4*, *CYP3A5*, *CYP3A7*, and *CYP3A43*, and this family is responsible for up to 50% of total CYP450 in the liver and the metabolism of ∼50% of all clinically relevant drugs [23,24,26,27]. There is little to no catalytic activity associated with *CYP3A43*, and the expression of *CYP3A5* is largely determined by the presence of the CYP3A5*1 allele [28] and additional SNPs are responsible for silencing this metabolic activity, so the examination of these two genes might not be as useful as other family members and will not be discussed further.

The expression of the *CYP3A* genes show a strong age dependency. During fetal development, *CYP3A7* is expressed. This enzyme has a role in the biosynthesis of estriol, which is involved in fetal growth and development, in the timing of parturition and the metabolism of endogenous compounds such as retinoic acid and dehydroepiandrosterone [25,29,30]. With the exception of a subset of individuals with a specific polymorphism that allows continued expression of *CYP3A7* in the postnatal period, in most individuals, the expression of *CYP3A7* declines in the first years after birth to the low adult levels. Concomitant with the decrease in *CYP3A7* expression, there is a progressive increase in the expression of *CYP3A4* from birth to 5 to 10 years of age, and mature levels of *CYP3A4* may not be reached until well into adolescence in some individuals [23,24,26,27]. The ORG HLC express not only high levels of *CYP3A7*, suggesting a fetal phenotype, but also levels of *CYP3A4* that exceed that observed in the fetal samples and overlap with the lower levels observed in mature liver, indicating maturation of this *CYP3A* family member beyond the fetal level.

The *CYP2C* family comprises ∼20% of the total CYP450 in the liver and is responsible for the metabolism of 20–30% of the clinically relevant drugs. During development, the expression of *CYP2C19* appears first and is the dominant *CYP2C* family member during most of the fetal period. *CYP2C9* activity progressively increases and becomes the dominant *CYP2C* family member at birth. Newborns may express up to 10% of the adult levels of *CYP2C9* and full adult levels are thought to be attained by 6 months of age [23–25]. Much less is known about *CYP2C8* expression and metabolic activity during development. In the ORG HLC, *CYP2C* family genes were expressed at, or slightly above, levels normally observed in fetal hepatocytes, and no *CYP2C* family members approached the expression observed in adults.

The Uridine 5'-diphospho-glucuronosyltransferase (UDP-glucuronosyltransferase, UGT) enzymes also show age-dependent changes in expression. These enzymes are responsible for the glucuronidation of many endogenous hydrophobic compounds such as bilirubin, bile acids, thyroxine, and exogenous compounds such as morphine, acetaminophen (paracetamol), and chlopamphenicol [23–25]. Conjugation of bilirubin, and SN-38, the active metabolite of the topoisomerase I inhibitor irinotecan is accomplished by *UGT1A1* [31]. Expression of *UGT1A1* is low to negligible during the fetal period, but there is a burst of activity at birth that is not dependent on gestational age. This observation suggests that postnatal factors, rather than gestational or developmental factors are responsible for the increase in expression and metabolic activity in the postnatal period. Morphine is conjugated by *UGT2B7*, and morphine conjugating activity follows a pattern similar to *UGT1A1*, with little activity during the fetal period and a burst of expression and activity in the postnatal period, and like *UGT1A1*, approaches adult levels by ∼6 months of age. The expression of *UGT1A1* and *2B7* in ORG HLC is higher than the levels in fetal liver and approaches the lower levels observed in adult liver samples. The expression of enzyme activity associated with *UGT1A6* and *UGT1A9* is also negligible at birth, but follows a slower developmental profile compared to *UGT1A1* and *UGT2B7*, with slower and a more progressive increase with postnatal age to adult levels by 1–10 years. What little is known about the temporal expression of *UGT2B10* and *UGT2B17* suggests that the expression of these family members is negligible in the fetal period and still less than 10% of adult levels in the neonatal period. The expression of these UGTs in ORG HLC exceeds that observed in fetal liver and approaches (*UGT1A6* and *UGT2B17*) or falls within adult levels (*UGT1A9*). In this study, no difference was observed in the expression of *UGT2B10* between fetal and older liver tissues.

Genes with a liver-enriched profile are, themselves, regulated by a number of liver enriched transcription factors. In particular, the *CYP2B*, *CYP2C*, *CYP3A*, and *UGT1A* family members are regulated, in part, by the expression of the *HNF* family, C/EBP, pregnane X receptor (*PXR*), and constitutive androstane receptor (*CAR*) and other nuclear hormone receptor transcription factors [27,32–36]. Failure to express the target genes at mature liver levels could result from lower than normal levels of these transcriptional activator genes. Members of the *HNF* family genes were expressed in ORG HLC at levels normally observed in mature liver, however, *PXR* was expressed at levels normally observed in fetal liver, suggesting that immature levels of this transcription factor may be, in part, responsible for some of the fetal characteristics of the *CYP2C* and *CYP3A* family members in iPSC-derived HLC. Although the expression of both *PXR* and *CAR* is low in the ORG HLC, the expression of *CAR* exceeds the median of the fetal samples and approaches the lower level of the mature liver samples. Since the expression of *CYP2B6* is, in part, dependent on *CAR* expression, this may explain why *CYP2B6* expression in ORG HLC is also higher than that observed in the fetal samples and approaches the lower levels of the mature liver samples.

Maybe somewhat surprising, many of the transcription factors and growth factor pathways known to regulate mature liver gene expression are expressed at or near normal mature liver levels in most of the ORG HLC, particularly *HNF1*, *HNF3*, *HNF4*, *HNF6*, *LXRa*, *FXR*, *PPARg*, *CTNNB1*, *AHR*, *GR*, and *MET*. These transcription factors and growth factor pathways are thought to be critical for hepatic differentiation. However, their expression seems to be required, but not sufficient for complete mature liver gene expression.

Concerning the *HNF* family, this study only measured gene expression, not transcriptional activity, so even though mature liver levels of the *HNF* family genes may be present in the ORG HLC, there is no information concerning the functional activity of the protein from these studies. There are different isoforms of *HNF4a* generated depending on the promoter region utilized combined with 3' splicing events. Transcripts driven from the P1 promoter are found in adult liver, colon, and pancreas, whereas transcripts driven from the P2 promoter are more abundant in fetal liver and in many epithelial cancers, including liver and colon [37–39]. Some studies suggest that the transition from P2 to P1 promoter driven transcripts parallels the transition from the fetal to adult phenotype in the liver [37]. Differential transcripts of *HNF4a* driven from the P1 and P2 promoters were analyzed, and as shown in Fig. 6, the pattern of expression of *HNF4a* isoforms in ORG HLC was similar to that observed in fetal liver and favored P2 promoter-driven transcripts, indicating that a more fetal-like phenotype could be expected in the ORG HLC for genes driven by this HNF4a isoform. The expression of other characteristic fetal or stem cell-related genes such as *CYP3A7*, *AFP*, *DLK1*, *KRT7*, *KRT19*, and *MRP4* in the HLC, at levels found in fetal liver clearly demonstrate that the cells have a fetal phenotype with respect to these genes.

Despite the expression of many fetal genes, by ORG HLC, they do not have a complete fetal phenotype, since many genes, including *CYP2B6*, *CYP3A4*, *CYP7A1*, *A1AT*, *PAH*, *FAH*, *CPS1*, *UGT1A1*, *UGT1A6*, *UGT1A9*, *UGT2B7*, *UGT12B17*, *NTCP*, *BSEP*, *MDR3*, and cytokeratins 8 and 18 and other genes, are expressed at levels higher than that observed in fetal liver, and approaching, or in some cases equaling, the level of expression observed in mature liver. The mixed expression of fetal genes with more mature genes suggests a pattern of differentiation of ORG HLC, ex vivo, that differs from that normally followed in liver during normal, in vivo, development. Modifications of culture and growth factor conditions to enhance *PXR* and P1-driven *HNF4a* expression would seem a logical approach to enhance the expression of stem cell-derived hepatocyte-like cells to a more mature phenotype.

There are many research groups focusing on hepatic differentiation of stem cells, but few have access to primary hepatocytes or human liver tissue that could serve as positive controls. Real-time RT-PCR is a low-cost and an accurate molecular biology technique that requires only basic laboratory skills, with machinery available in almost all laboratories. Therefore, the gene expression data from primary fetal and mature liver tissues assembled in this study should be useful and convenient for the assessment of hepatic differentiation protocols. Whole transcriptome sequencing would provide more information, but at a much higher cost, and it demands more advanced bioinformatics skills. It is understood that the present studies only investigate gene expression, but not actual protein expression or function, and the correlation between these two can differ for some genes [40]. Other methods for protein quantification could have been applied, such as Enzyme-Linked Immunosorbent Assay, mass-spectrometry or immunocytochemistry, or quantification of metabolic activity of phase I and phase II metabolizing enzymes [41] could add additional information. These techniques are more complex and are more difficult to standardize and compare between laboratories. Most importantly, they require far more cells than RT-qPCR studies and are not sufficiently high-throughput in most laboratories for screening purposes. For efficiency and cost-effectiveness, functional and metabolic studies should be planned as a second tier study when rapid screening with the protocol described here reveals that the HLC express meaningful levels of the genes(s) of interest.

The hepatic tissues included in this study are from individuals of different ages, ranging from 74 days to 24 weeks for the fetal samples and from 5.5 to 79 years for the postnatal tissues. Interindividual variability has been reported in liver and cultured hepatocytes [31,41,42]. We also observed wide variability in gene expression between tissues from different individuals in this study, especially for the CYP genes which are known to be highly inducible by xenobiotic exposure, where there was a four-log wide range in values for some CYPs. These results reinforce the need for the inclusion of controls from more individuals when assessing the efficacy of a differentiation protocols for iPSC-HLC. In most differentiation studies, one or only a few fetal or/and adult tissues [19,43] or cancer cell lines, such as HepG2, are used as controls [44]. Intraindividual variability and low numbers of liver samples could easily lead to inaccurate conclusions concerning the state of differentiation of the HLC.

In conclusion, generation of HLC from stem cell sources is a current challenge in the regenerative medicine field. The aim of the study was to provide a simple procedure that could be performed in nearly every laboratory to assess hepatic maturation of HLC. To our knowledge, there is no differentiation protocol that produces HLC capable of conducting the majority of the hepatic functions at a level equal to adult liver. Further optimization of differentiation protocols is necessary, which will require quick and accurate assessment of the efficacy of the differentiation procedure. The protocol proposed in this study to examine gene expression by quantitative RT-PCR is simple, low-cost and requires only basic laboratory skills and equipment. In addition, a robust data set generated from 42 fetal or mature human liver tissues is provided and characterized so that the gene expression in any HLC sample could quickly and easily be compared to that observed in fetal and mature human liver.

## Financial Support

This research was supported by CIMED, Clinical Innovative Medicine, Ventenskaprådet (Swedish Research Council) European Commission, EU/FP7, HUMAN and the Torsten och Ragnar Söderberg Stiftelse (SCS). This work was supported by grants from NIH, DK099257 and DK117881 to A.S.-G., and by the American Liver Foundation and the Uehara Memorial Foundation to K.T.

## Supplementary Material

Supplemental data

Supplemental data

Supplemental data
