# Supplementary material for: Guide to the Assessment of Mature Liver Gene Expression in Stem Cell-Derived Hepatocytes
Source: Stem Cells Dev. 2019 Jul 16;28(14):907–19. doi: 10.1089/scd.2019.0064 (PMC6648222; doi:10.1089/scd.2019.0064)
Supplement: Supplemental data [file Supp_TableS2.pdf]

Supplementary Table S2. TaqMan Assays Used for Gene Expression Analysis

	Gene symbol	*Gene name*	*Assay ID*
	PPIA	Cyclophilin A (peptidylprolyl isomerase A)	Hs99999904_m1
Liver-specific plasma proteins/metabolic genes	ALB	Human albumin	Hs00609411_m1
	AFP	Alpha-fetoprotein	Hs00173490_m1
	A1AT	Alpha-1 antitrypsin (SERPINA1)	Hs01097800_m1
	GLUL	Glutamate-ammonia ligase (glutamine synthetase)	Hs01018343_g1
	OTC	Ornithine carbamoyltransferase	Hs00166892_m1
	CPS1	Carbamoyl-phosphate synthetase 1	Hs00157048_m1
	PAH	Phenylalanine hydroxylase	Hs00609359_m1
	FAH	Fumarylacetoacetate hydrolase	Hs00908445_m1
Phase I genes	CYP1A1	Cytochrome P450 family 1 subfamily A member 1	Hs00153120_m1
	CYP1A2	Cytochrome P450 family 1 subfamily A member 2	Hs01070374_m1
	CYP2B6	Cytochrome P450 family 2 subfamily B member 6	Hs03044634_m1
	CYP2C8	Cytochrome P450 family 2 subfamily C member 8	Hs00258314_m1
	CYP2C9	Cytochrome P450 family 2 subfamily C member 9	Hs00426397_m1
	CYP2C19	Cytochrome P450 family 2 subfamily C member 19	Hs00426380_m1
	CYP2D6	Cytochrome P450 family 2 subfamily D member 6	Hs02576167_m1
	CYP3A4	Cytochrome P450 family 3 subfamily A member 4	Hs00430021_m1
	CYP3A7	Cytochrome P450 family 3 subfamily A member 7	Hs00426361_m1
	CYP7A1	Cytochrome P450 family 7 subfamily A member 1	Hs00167982_m1
	CYP7B1	Cytochrome P450 family 7 subfamily B member 1	Hs00191385_m1
Phase II genes	UGT1A1	UDP glucuronosyltransferase 1 family member A1	Hs02511055_s1
	UGT1A6	UDP glucuronosyltransferase 1 family member A6	Hs01592477_m1
	UGT1A9	UDP glucuronosyltransferase 1 family member A9	Hs02516855_sH
	UGT2B7	UDP glucuronosyltransferase 2 family member B7	Hs00426592_m1
	UGT2B10	UDP glucuronosyltransferase 2 family member B10	Hs02556282_s1
	UGT2B17	UDP glucuronosyltransferase 2 family member B17	Hs00854486_sH
Transcriptions factors	LXRα	Liver X receptor alpha (NR1H3)	Hs00172885_m1
	LXRβ	Liver X receptor beta (NR1H2)	Hs01027215_g1
	FXR	Farnesoid X nuclear receptor (NR1H4)	Hs00231968_m1
	PXR	Pregnane X nuclear receptor (NR1I2)	Hs01114267_m1
	CAR	Constitutive androstane nuclear receptor (NR1I3)	Hs00231959_m1
	PPARα	Peroxisome proliferator-activated receptor alpha (NR1C1)	Hs00947536_m1
	PPARγ	Peroxisome proliferator-activated receptor gamma (NR1C3)	Hs01115513_m1
	HIF1A	Hypoxia-inducible factor 1-alpha	Hs00153153_m1
	HNF1a	Hepatic nuclear factor 1 alpha	Hs00167041_m1
	HNF1b	Hepatic nuclear factor 1 beta	Hs00172123_m1
	HNF3a	Hepatic nuclear factor 3 alpha (FOXA1)	Hs04187555_m1
	HNF3b	Hepatic nuclear factor 3 beta (FOXA2)	Hs00232764_m1
	HNF4a	Hepatic nuclear factor 4 alpha	Hs00230853_m1
	HNF4a–P1 promoter-drived–Adult isoforms	Hepatic nuclear factor 4 alpha	Hs00604431_m1
	HNF4a–P2 promoter-drived–Fetal isoforms	Hepatic nuclear factor 4 alpha	Hs01025522_m1
	HNF6	Hepatic nuclear factor 6 (ONECUT1)	Hs00413554_m1
	AHR	Aryl hydrocarbon receptor	Hs00169233_m1
	GR	Glucocorticoid receptor (NR3C1)	Hs00353740_m1
Transporters	NTCP	Sodium/bile acid cotransporter 1 (SLC10A1)	Hs00914889_m1
	P-GP	ATP-binding cassette, B1 (ABCB1 - MDR1)	Hs00184500_m1
	MDR3	ATP-binding cassette, B4 (ABCB4)	Hs00240956_m1
	MRP2	ATP-binding cassette, C2 (ABCC2)	Hs00166123_m1
	MRP3	ATP-binding cassette, C3 (ABCC3)	Hs00978473_m1
	MRP4	ATP-binding cassette, C4 (ABCC4)	Hs00988717_m1
	BSEP	ATP-binding cassette, B11 (ABCB11)	Hs00184824_m1
	BCRP	ATP-binding cassette, G2 (ABCG2)	Hs00184979_m1
Pluripotency genes	DLK1	Delta-like 1 homolog	Hs00171584_m1
	NANOG	Nanog homeobox	Hs04260366_g1
	SOX2	SRY (sex determining region Y)-box 2	Hs01053049_s1
	SOX9	SRY (sex determining region Y)-box 9	Hs01001343_g1
	SOX17	SRY (sex determining region Y)-box 17	Hs00751752_s1
	OCT4	Octamer-binding transcription factor	Hs00742896_s1

(*continued*)

Supplementary Table S2. (Continued)

	Gene symbol	*Gene name*	*Assay ID*
Other genes	KRT7	Keratin 7 (KRT7)	Hs01115174_mH
	KRT8	Keratin 8 (KRT8)	Hs02339474_g1
	KRT18	Keratin 18 (KRT18)	Hs01653110_s1
	KRT19	Keratin 19 (KRT19)	Hs00761767_s1
	CTNNB1	CTNNB1, cadherin-associated protein	Hs00355049_m1
	MET	Met proto-oncogene (HGF receptor)	Hs01565582_g1
	ASGR1	Asialoglycoprotein receptor 1	Hs00155881_m1
