# Supplementary material for: Guide to the Assessment of Mature Liver Gene Expression in Stem Cell-Derived Hepatocytes
Source: Stem Cells Dev. 2019 Jul 16;28(14):907–19. doi: 10.1089/scd.2019.0064 (PMC6648222; doi:10.1089/scd.2019.0064)
Supplement: Supplemental data [file Supp_TableS1.pdf]

Supplementary Data

Supplementary Table S1. Information on the Donor Tissues

Tissue	*Sample ID*	*Age*	*Gender*
Fetal	1	16 weeks	UN
Fetal	2	17 weeks	UN
Fetal	3	22 weeks	UN
Fetal	4	17 weeks	UN
Fetal	5	91 days	M
Fetal	6	22 weeks	M
Fetal	7	20 weeks	UN
Fetal	8	21 weeks	UN
Fetal	9	19 weeks	F
Fetal	10	16 weeks	UN
Fetal	11	24 weeks	UN
Fetal	12	89 days	M
Fetal	13	88 days	M
Fetal	14	89 days	F
Fetal	15	91 days	F
Fetal	16	89 days	M
Fetal	17	74 days	M
Mature	18	12 years	F
Mature	19	62 years	M
Mature	20	44 years	M
Mature	21	18 years	F
Mature	22	15 years	F
Mature	23	28 years	F
Mature	24	70 years	M
Mature	25	64 years	M
Mature	26	43 years	F
Mature	27	23 years	F
Mature	28	25 years	F
Mature	29	67 years	F
Mature	30	70 years	M
Mature	31	57 years	M
Mature	32	44 years	M
Mature	33	22 years	M
Mature	34	79 years	M
Mature	35	57 years	M
Mature	36	49 years	F
Mature	37	16 years	M
Mature	38	12 years	F
Mature	39	30 years	F
Mature	40	5,5 years	M
Mature	41	24 years	M
Mature	42	53 years	M
